# Miscellany

**Published:** 1912-07

**Authors:** 


### MISCELLANY

The U. S. Government has forbidden the importation of absinthe after Jan. 1, 1812 on account of the demonstrated harmfulness of its use as a beverage. A recent bulletin of the Dept. of Agriculture, describing native plants of medicinal virtues, with the idea of stimulating the industry of collecting them, lists absinthe as having been early introduced into this country and as now being a natural product. Absinthe is a fairly useful drug of the old-fashioned kind but, obviously, something more than prohibiting its importation will be required to prevent its use as a tipple.

There are now 400,000 automobiles in the country, as against 3,500 for 1901. More than a billion dollars has been paid for them in ten years and their cost, both initially and in up-keep, exceeds that of our navy, which it is the fashion to denounce as an extravagance. About one family in 40 has an automobile. Nowhere near this proportion felt that they could afford a carriage and team. The mileage and time spent in transit has been enormously increased. Many amateur economists claim that the automobile is responsible for our hard times. On the other hand, the money liberated by deaths caused by automobiles which affects mostly the well-to-do class numbering approximately one two-hundredth of the population, has probably acted to postpone financial stringency. The automobile mortality, by the way, is almost exactly equal to that caused by railroads and street cars together, although the part of the population affected is very much smaller.

### Yellow Medicine

Motion pictures will be used to further the anti-tuberculosis campaign. One of these is about as follows: The villain is a heartless boss, in whose neglected tenements, a number of cases arise. His wife contracts the disease from nursing one of these on ship board. There is no room for her in any private sanitarium. (Here would be a good place to throw on the screen the advertisements of a dozen or more excellent ones. Ed.) The distracted husband tries to bribe the secretary of the society to make a place for her but there is no public institution because the boss has voted against it. (How about Raybrook and others?) The bribe is accepted, however, and a bunch of red cross stamps handed over. The husband is angry. (We don't blame him.) But he is later touched when the work of the association is explained to him—$150,000. Then he is shown a tuberculosis exhibit and everything ends happily with the wife and tenement house children restored to health and the boss's candidate supported because of his pledge to the establishment of a tuberculosis hospital.

We are most heartily in favor of the work to limit tuberculosis and believe thoroughly in education of the masess but we can't enthuse over this kind of cheap cinemetograph vaudeville. In the long run, truth and dignity are better weapons than exaggeration, graft and appeals to prejudice.

---

Wisner R. Townsend, New York City. *New York State Journal of Medicine,* June, 1912, quotes the following statistics of INFANTILE PARALYSIS from the Massachusetts Report of 1910, analysing 200 cases

| Time of intitial Paralysis. | Cases. |
|---|---|
| Same day | 20 |
| One day | 31 |
| Two days | 40 |
| Three days | 34 |
| Four days | 15 |
| Five days | 11 |
| Six days | 11 |
| Seven days | 14 |
| | 176 |

the balance occuring at different periods up to the eighth week. The permanency and the extent of the paralysis are also matters of great importance. The early paralysis may involve many muscles which subsequently recover more or less completely or the primary paralysis may involve a few muscle groups or single muscles which may or may not remain paralyzed. Cases where the paralysis appears in one portion of the body at one time and in another at a subsequent time are infrequent; as a rule the primary paralysis is more extensive than the permanent.

Type of deformity:

| | |
|---|---|
| Drop foot | 6 |
| Genu recurvatum | 25 |
| Talipes varus | 3 |
| Talipes eq. varus | 3 |
| Talipes valgus | 9 |
| Talipes calcaneus | 7 |
| Talipes calcaneo-valgus | 2 |
| Talipes equinus | 14 |
| Lateral curvature | 2 |
| Drop wrist, etc. | 1 |
| Weakness abdominal muscles | 3 |
| Right valgus / Left calcaneus | 1 |
| Right drop / Left valgus | 1 |
| Multiple deformities | 3 |

| Distribution of early paralysis: | Cases. |
|---|---|
| One leg only | 145 |
| Both legs only | 146 |
| One arm only | 44 |
| Both arms only | 12 |
| One arm and leg, same side | 50 |
| One arm and leg, opposite sides | 18 |
| Both legs and one arm | 32 |
| Both arms and one leg | 8 |
| Both arms and both legs | 51 |
| Ataxia (transitory) | 7 |
| Back | 79 |
| Abdomen | 38 |
| Neck | 13 |
| Respiration | 39 |
| Deglutition | 12 |
| Intercostal | 1 |
| Face | 7 |
| Right face | 31 |
| Left face | 24 |
| Strabismus | 2 |
| Not stated | 32 |

